# Circumferential silicone sponge scleral buckling induced axial length changes: case series and comparison to literature

**DOI:** 10.1186/s40942-017-0063-1

**Published:** 2017-03-27

**Authors:** Shira Sheen Ophir, Asaf Friehmann, Alexander Rubowitz

**Affiliations:** Department of Ophthalmology, Meir Medical Center, 59 Tshernichovsky St., Kfar Saba, 44281 Israel

**Keywords:** Silicone sponge, Solid silicone rubber, Axial length, Circumferential buckling, Rhegmatogenous retinal detachment

## Abstract

**Background:**

This study compared axial length changes induced by circumferential scleral buckling using a silicone sponge with literature reports for solid silicone rubber.

**Methods:**

Records of patients treated with scleral buckling in 2009–2013 using a silicone sponge, with preoperative axial length biometry measurements were reviewed. Additional information included age, type of surgery, additional surgeries, phakic status and anatomical success of reattachment. Patients underwent repeat biometry. The medical literature was reviewed for articles describing axial length changes induced by circumferential buckling using solid silicone rubber.

**Results:**

Twenty-eight patients (mean age 49.7 years, range 16–72) met the inclusion criteria. Mean axial length was 25.38 mm preoperatively and 26.12 mm at least 6 months postoperatively (SD 0.50 ± 0.09, *p* < 0.001); a mean increase of 0.74 mm. Half the patients subsequently underwent cataract surgery. Post-operative changes were not significant compared to pre-surgical refraction and corneal astigmatism. Axial length change was not significant between sexes (9 women and 19 men).

**Conclusions:**

Axial length changes induced by circumferential scleral buckling using silicone sponge exclusively are similar to those reported in the literature for solid silicone rubber buckles. Scleral buckling using a silicone sponge, which may offer several surgical advantages, induces an acceptable axial length change similar to that seen with widely-used solid silicone rubber buckles.

## Background

Although its popularity has declined in recent years [1], scleral buckling is still commonly used for the treatment of rhegmatogenous retinal detachment, either as a stand-alone procedure or combined with vitrectomy. Scleral buckling has several drawbacks and unique side effects, including conjunctival scarring, explant exposure or infection, diplopia (usually transient), globe penetration while suturing the explants to the sclera, and others [2–6]. Axial length change, one of the most common side effects of scleral buckling surgery, is caused by the buckle deforming and indenting the globe, which causes a refractive shift. This common side effect is particularly bothersome in previously emmetropic patients, in patients previously isometropic in whom it may induce anisometropia, and in previously emmetropic pseudophakic patients, who are frustrated by the need for refractive correction in a previously ametropic eye after cataract surgery.

Scleral buckling using solid silicone rubber explants is the most widely used form of buckling surgery, and several publications have documented the axial length changes it induces [7–18]. Our department uses only silicone sponge explants, rather than the solid silicone rubber explants used in most institutions. To the best of our knowledge, axial length changes induced exclusively by using silicone sponge buckles have not been well documented. In this study, we sought to determine whether the change in axial length induced by silicone sponge circular explants was significantly different from that reported in the literature for solid silicone explants, and whether one explant material was more advantageous than the other was.

## Methods

Records of all patients who underwent scleral buckling surgery in our department during 2009–2013 by two vitreoretinal senior surgeons (Y.F. and A.R.) using circumferential 7.5 mm × 2.5 mm silicone sponge explants (Labtician Ophthalmics, Style 511, Oakville, ON, CA) were reviewed. Scleral sutures were placed at 9 mm width (1.5 mm wider than the sponge), using 5–0 Ethilon (Ethicon US, Somerville NJ, USA) scleral mattress sutures to provide an indentation effect.

Only patients with a successfully attached retina at least six months after surgery, whose charts contained reliable preoperative axial length measurements using the Zeiss IOL Master (Carl Zeiss Meditech, Jena, Germany), without intravitreal silicone oil (which would preclude reliable axial length measurement), and who had circumferential explants placed without additional radial buckling elements were included. Patients with these characteristics were called and invited to participate in the study. Those who chose to participate received an explanation of the study goals and signed an informed consent. Subsequently, they underwent at least three repeated IOL-Master biometry axial length measurements, the axial length measurements were performed by two ophthalmologists (S.S.O. and A.F.)

The charts were reviewed for surgical details, preoperative phakic status, and demographic data such as age and sex. The pre- and post-operative biometrics were compared using paired *t* test. *p* < 0.05 was considered statistically significant. Statistical analyses were performed using SPSS, Inc., version 21.

As approximately half the patients were phakic at the time of their detachment surgery and later had cataract surgery, their refractive status was influenced by differing techniques and intraocular lens types and was therefore not included in the analysis.

The literature was reviewed for articles describing axial length changes caused by circumferential scleral buckling with solid silicone rubber explants or silicone sponge explants, including articles in all languages from 1970 to the present.

## Results

Twenty-eight eyes of 28 patients met the inclusion criteria. Mean age was 49.7 years (range 16–72 years). The mean axial length was 25.38 mm preoperatively, and 26.12 mm at least 6 months postoperatively (SD 0.497 ± 0.094, *p* < 0.001), a mean increase of 0.74 mm (Table 1).

**Table 1:** Pre- and postoperative measurements of 28 eyes

Measurement	Preoperative	Postoperative	Std. Deviation	*p* value
Axial length (mm)	25.38	26.12	0.497 ± 0.094	<0.001
Refraction (diopters)	−2.78	−3.49	2.36 ± 0.52	0.17
Cylinder (diopters)	−1.03	−1.39	0.75 ± 0.14	0.17

Refraction did not change significantly after surgery (−2.78D before to −3.49 after, SD 2.58 ± 0.58, *p* = 0.156, respectively). Axial length change between sexes was not significant (9 women and 19 men). However, more males underwent surgery.

Corneal astigmatism did not change significantly after surgery (−1.03D to −1.39D respectively, SD 0.75 ± 0.14, *p* = 0.17).

## Discussion

Axial length change is a common side effect of scleral buckling surgery. It is caused by the buckle causing an indentation in the globe, which might cause a refractive shift. Our ophthalmology department uses soft, pliable, silicone sponge buckles, as we feel they induce a more gradual and rounded indentation of the globe wall than that seen with solid silicone buckling. We compared this method with the use of solid silicone reported in the literature.

Although numerous articles describe axial length changes after scleral buckling surgery [7–17], the surgeries and methods used, as well as patient characteristics, length of follow-up and measurements reported vary widely. This makes it difficult to establish a standard, acceptable axial length change with the commonly used method of solid silicone rubber buckling, and complicates comparison with the buckling method used in our institution.

Some reports regarding axial length change after scleral buckling used the older method of intra-scleral implants rather than the explants used today [8–10, 14]. Others included several methods in undisclosed proportions or various surgical methods that were not described [8, 9, 15, 16, 18], also precluding meaningful comparison.

Sato et al. [14] included only pediatric eyes. This causes additional comparison problems due to the possibility of differing effects of buckling on globe growth and scleral compliance at various ages. Additionally, one article included buckle height measurements but not axial length [9], while others measured axial lengths after very short or varying and unspecified periods of follow-up [10, 17], where the eyes may not have reached their final axial length. Most of these articles included several of the above mentioned limitations.

Previously published articles used A-scan ultrasound to measure axial length, whereas we used optical biometry with the Zeiss IOL-Master. Optical biometry has been shown to yield extremely accurate and reliable axial length measurements, and measurements obtained with these two methods agree closely and are interchangeable [19–23].

The few remaining articles, where exclusively solid silicone buckles and elements were used as explants, with methods described in detail, adequate follow-up and axial length measurements were used as a comparison to our buckling method. Larsen and Syrdalen [11] found that the average axial length increased 0.98 mm in 10 eyes, Brazitikos et al. [7] reported a mean axial length increase of 0.95 mm in 75 eyes and Malukiewicz-Wisniewska and Stafiej [12] showed a mean increase at one year of 0.57 mm in all 74 eyes. As can be seen in the relevant articles, the mean axial length increased from 0.57 mm to 0.98 mm after scleral buckling surgery.

## Conclusion

We found a mean axial length increase of 0.74 mm at least 6 months after surgery. This is comparable (although possibly closer to the lower end) to that previously described for scleral buckling with solid silicone elements.

We did not find any reports describing axial length changes after scleral explant surgery using a silicone sponge. Although, several papers described axial length changes with combined solid silicone and silicone sponge elements.

In this study, we have shown that axial length changes six months after scleral explant surgery using a silicone sponge are comparable to those described in the literature after solid silicone rubber buckling.
